# A Newly Emerging Serotype A Strain in Foot-and-Mouth Disease Virus with Higher Severity and Mortality in Buffalo than in Cattle Calves in North Egypt

**DOI:** 10.3390/vetsci10080488

**Published:** 2023-07-27

**Authors:** Samy Metwally, Nabil Bkear, Yassien Badr, Besheer Elshafey, Sadeq K. Alhag, Laila A. Al-Shuraym, Gaber Batiha, Bassant Fakhry, Rania Hamada

**Affiliations:** 1 Division of Infectious Disease, Department of Animal Medicine, Faculty of Veterinary Medicine, Damanhour University, Damanhour 22511, Egypt; nabil.baker@vetmed.dmu.edu.eg (N.B.); yaseen.badr@vetmed.dmu.edu.eg (Y.B.); bassantbassant95@gmail.com (B.F.); 2 Laboratory of Global Infectious Diseases Control Science, Graduate School of Agricultural and Life Sciences, The University of Tokyo, Bunkyo-Ku, Tokyo 113-8657, Japan; 3 Division of Internal Medicine, Department of Animal Medicine, Faculty of Veterinary Medicine, Damanhour University, Damanhour 22511, Egypt; besheer_elshafey@vetmed.dmu.edu.eg; 4 Biology Department, College of Science and Arts, King Khalid University, Muhayl Asser 61913, Saudi Arabia; salhag@kku.edu.sa; 5 Biology Department, Faculty of Science, Princess Nourah Bint Abdulrahman University, Riyadh 11671, Saudi Arabia; laalshuraym@pnu.edu.sa; 6 Department of Pharmacology and Therapeutics, Faculty of Veterinary Medicine, Damanhour University, Damanhour 22511, Egypt; gaber_saber@dmu.edu.eg; 7 Division of Clinical Pathology, Department of Pathology, Faculty of Veterinary Medicine, Damanhour University, Damanhour 22511, Egypt

**Keywords:** FMDV, serotype A, buffalo, calves, fatal, Egypt

## Abstract

**Simple Summary:**

Foot-and-mouth disease virus (FMDV) is the etiologic agent of foot-and-mouth disease (FMD), which causes severe economic losses among cloven-hoofed animals in endemic nations. Egypt is a bridge for FMDV pools between continents, where three serotypes (A, O, and SAT2) have established an enzootic state. Recently, a severe FMD epidemic struck several farms in the Beheira province, north Egypt. This study was the first to detect the newly emerging FMDV, serotype A, Europe–South America (Euro–SA) topotype that was collected during such outbreaks in twenty calves’ tongue epithelial samples from five infected farms based on virus isolation, molecular methods, and phylogenetic analysis. Additionally, we found that this strain caused higher peracute mortalities in water buffalo (*Bubalus bubalis*) (25.7%; 95% CI: 13–43) than in cattle (8.6%; 95% CI: 2–24) calves. Meanwhile, in fatal cases, severe clinical signs such as dullness, hypothermia, bradycardia, and cardiac arrhythmia were common in both species. In conclusion, we first characterized the newly emerging FMDV in the calves of Beheira as more fatal and severe in buffalo than in cattle calves.

**Abstract:**

A severe foot-and-mouth disease (FMD) epidemic struck several Egyptian provinces recently, causing significant losses among animals even in vaccinated farms. This study indicated the existence of the newly emerging foot-and-mouth disease virus (FMDV) and first investigated its effect on the Egyptian water buffalo (*Bubalus bubalis*) and cattle calves in the Beheira province, north Egypt. Twenty tongue epithelial samples from diseased calves in five infected farms were randomly collected, prepared, and propagated using baby hamster kidney-21 (BHK-21) cells. Whole genomic RNA was extracted from the cells of the third passage. A FMDV genome was detected and serotyped using one-step reverse transcription polymerase chain reactions (RT-PCRs). Nucleotide sequencing of the purified serotype-specific PCR bands was performed, and a maximum likelihood phylogenetic tree based on 600 base pairs of VP1 was constructed. The results identified FMDV, serotype A in all infected samples, whereas the serotypes O and SAT2 were negative. The obtained 20 sequences were identical to each other and similar to the newly reported strain in Egypt that belongs to the Europe–South America (Euro–SA) topotype. The epidemiological and clinical parameters associated with such a strain were fully recorded by veterinarians and analyzed in a single infected farm including 70 cattle and buffalo calves. It caused higher peracute mortalities in buffalo (25.7%; 95% CI: 13–43) than in cattle (8.6%; 95% CI: 2–24) calves. Severe clinical signs such as dullness, hypothermia, bradycardia, and cardiac arrhythmia were common to both except in fatal cases, whereas hyperthermia and respiratory signs were prevalent in cattle calves. In conclusion, we first characterized the newly emerging FMDV in the calves of Beheira as more fatal and severe in buffalo than in cattle calves.

## 1. Introduction

Foot-and-mouth disease (FMD) is a highly contagious disease in domestic, cloven-hoofed animals including cattle, buffaloes, goats, sheep, pigs, and more than 70 wild animal species [1,2]. It is caused by the foot-and-mouth disease virus (FMDV), a small, non-enveloped, single-stranded RNA virus belonging to the *Aphthovirus* genus of the family *Picornaviridae* [3]. FMD is regarded as the main cause of economic loss in the dairy and beef industries in endemic nations, as well as the main obstacle to international trade in animals and animal products [4,5,6]. It is characterized clinically by fever and vesicle formation on the mouth, muzzle, teats, and feet, which causes ropy salivation, difficult mastication, and lameness. Morbidity in a population at risk was close to 100%, and young animals have significant mortality rates [4,7,8]. In calves, myocarditis is considered a fatal form of FMD that may occur without developing the characteristic vesicular lesions [8,9,10].

Seven immunologically different serotypes of FMDV, namely A, O, C, Asia 1, SAT 1, SAT 2, and SAT 3 exist, which are subdivided into a diverse number of topotypes, lineages, and sublineages [11,12,13,14]. The FMDV particle is icosahedral in shape, with a genome that is approximately 8400 nucleotides long and surrounded by a capsid protein coat [15]. The viral genome is divided into P1, P2, and P3 regions. The P1 region encodes the structural proteins, 1D (VP1), 1B (VP2), 1C (VP3), and 1A (VP4) [15], while the P2 and P3 regions encode non-structural proteins, protease, and polymerase [16]. Viral protein 1 (VP1) is responsible for virus attachment and entry, and protective immunity. It is highly polymorphic, and its nucleotide sequence is considered a golden standard for FMDV serotyping [15,17].

Globally, FMDV endemic regions have been classified into seven geographical pools [12,18,19]. In Egypt, three FMDV serotypes (A, O, and SAT2) have established an enzootic state [8,13,14,20,21]. FMDV, Serotype O was first reported in 1951 and serotype A was detected in 1972 [22]. Thirteen successive outbreaks of FMDV serotypes O and A were recorded between 1958 and 2000 [8,22]. Thirty-four FMD, serotype A outbreaks have been reported in eight Egyptian provinces in 2006 with a high genetic similarity to East African FMDV strains [23]. In 2012, infection with the newly emerged FMD, serotype SAT2 caused massive losses in cattle and buffaloes in several provinces [24]. Then, the emergence of FMDV, serotype SAT2, Lib-12 lineage of topotype VII caused an outbreak in 2018 [25]. A recent study in 2022 detected FMDV, serotype O, the Europe–South America topotype [13]. Additionally, FMDV, serotype A, the Europe–South America topotype, was detected [14].

The Egyptian FMD control program relies on mass vaccination of susceptible animals using multivalent vaccines [26]. Therefore, routine detection of the circulating serotypes is necessary [2,12,26]. Diagnosis of FMDV depends on cell-based methods, detection of specific antibodies, and molecular-based approaches using PCR and nucleotide sequencing [2,14,15,27].

Since the beginning of 2022 through the time of this analysis in May 2022, several FMD outbreaks have been observed in various provinces in Egypt. However, there was a lack of scientific research to identify the circulating serotypes. In July 2022, FMDV, serotype A, Euro-SA lineage had been first reported in a regular survey in Egypt [14]. However, the origin of this strain, how it was introduced, and its spread and virulence among Egyptian animals were poorly investigated. Therefore, this study was conducted to detect whether this strain was the causative agent during such outbreaks in the Beheira province. Additionally, we aimed to monitor the epidemiological patterns and the clinical findings associated with the circulating serotype in young buffalo (*Bubalus bubalis*) and cattle calves in a single infected herd based on the data recorded by the veterinarians and the farm’s records.

## 2. Materials and Methods

### 2.1. Ethical Approval

This research has obtained the approval of the Ethics of the Institutional Committee of the Faculty of Veterinary Medicine, Damanhour University, Egypt (DMU/VetINF-2022-/0148).

### 2.2. Study Population and Clinical Findings

**Figure 1:**
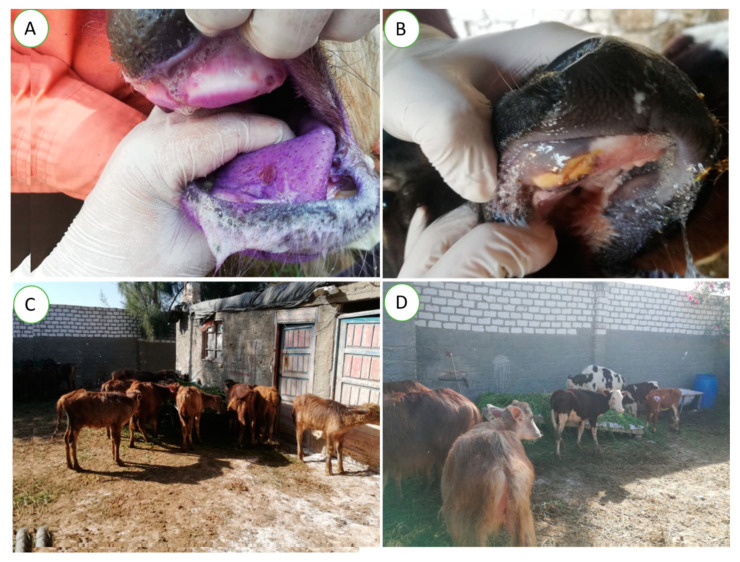
Calves showed clinical signs of foot-and-mouth disease: vesicles, erosions, and ulcers on the tongue and gums, and salivation in (**A**) buffalo calf that showed a violet discoloration of the oral cavity due to the topical use of antiseptics, (**B**) cattle calf. (**C**) Buffalo calves showed inappetence to anorexia, dullness, and isolation from the herd. (**D**) Cattle calves showed mild anorexia and dullness.

At the beginning of May 2022, several cattle and buffalo farms in the Beheira province suffered from outbreaks of FMD. Therefore, we randomly collected 20 tongue epithelial samples from 20 (10 cattle and 10 buffalo) infected calves in five infected non-vaccinated farms. These farms include both cattle and water buffalo (*Bubalus bubalis*) calves with ages of less than one year distributed in four districts in the Beheira province, namely, Damanhour, Abu Hommus, Abu Almatamer, and Kafr El-Dawar (Table 1). Regarding the study of clinical and epidemiological parameters associated with FMDV infection, calves from an infected farm in the Abu Almatamer district were fully investigated by veterinarians. In detail, a mixed semi-intensive farm comprised of 70 female animals at 4–6 months of age (35 buffalo and 35 cattle) showed a sudden appearance of clinical symptoms of FMD among the calves. Noticeably, these animals were purchased from the animal markets of Beheira, three months before the onset of clinical symptoms without a history of previous vaccination against FMD. During the outbreak, all calves suffered from high fever, salivation, lameness, vesicular eruption, ulcers on the tongue, gum, and coronets with different degrees of severity (Figure 1), and mortalities in both species. The clinical status and parameters of calves were observed by the veterinarians during the outbreak and for two months after the disappearance of the clinical symptoms. Noticeably, all data and clinical signs shown in this study were obtained from the veterinarians, owners, and the farm’s records.

### 2.3. Virus Sampling and Preparation

According to the standard measurements of the World Organization of Animal Health (WOAH) [28], 20 sloughed tongue epithelium samples were randomly collected from 20 infected animals (10 buffalo and 10 cattle). The specimens were mixed with equal amounts of glycerin in 0.04 M of phosphate buffer pH 7.2 [29] and transported into the BSL-2 laboratory (Veterinary Serum and Vaccine Research Institute, Cairo, Egypt). Each specimen was homogenized manually in Eagle’s minimum essential medium (MEM) (Sigma-Aldrich, New York, NY USA, followed by centrifugation at 2000× *g* for 20 min, then filtrated using syringe filters (0.2 µm). The supernatants were mixed with an antibiotic mixture of 1% (100 IU/mL of Penicillin and 100 mg/mL of Streptomycin). A part of each sample was subjected to a virus propagation experiment, and the other part was stored at −80 °C.

### 2.4. Virus Propagation and Cytopathic Effect Monitoring

The baby hamster kidney-21 cell lines (BHK-21 Clone 13, adapted in VSVRI, Cairo, Egypt) were used for virus propagation and CPE monitoring as previously described [14]. Three successive passages with daily monitoring of the CPE were carried out. Furthermore, a culture flask containing BHK-21cells was used as a negative control without sample inoculation.

### 2.5. Whole Genomic RNA Extraction and Molecular FMDV Genomic Detection

The infected BHK-21 cells of the third passage were harvested. BHK-21 cells were lysed via three successive freezing and thawing cycles and centrifuged at 1200× *g* for 20 min, then the supernatant was filtrated using syringe filters (0.2 µm). Whole genomic RNA was extracted using the EasyPure viral-RNA kit (TransGen Biotech, Beijing, China) according to manufacturer instructions. FMDV genomic RNA was investigated in the extracted RNA samples via RT-PCR using the pan-FMDV primers/probe set as described [30]. The RNA reverse transcription step was included in the amplification cycle (45 °C for 25 min) according to the manufacturer instructions of the TransScript^®^ Probe one-step qRT-PCR SuperMix (TransGen, Beijing, China). The PCR thermal cycle was performed on a BAX Q7 cycler (BAX Q7 systems, Marsiling, Singapore) as described previously [14].

### 2.6. FMDV Serotyping Using A-, O-, and SAT2-Specific Primers Targeting the 1D Gene

One-step RT-PCR was used to detect the existing FMDV serotype in the extracted RNA using serotype-specific PCR amplification of the VP1 protein genomic region. The used primer sets were amplifying 814, 1124, and 666 bp of the 1D variable gene of serotypes A, O, and SAT2, respectively, as described previously [21]. The reaction was carried out using the EasyScript^®^ one-step RT-PCR SuperMix (TransGen, Beijing, China) according to manufacturer instructions. The thermal conditions were adjusted as described previously [14]. The PCR products were documented via UV rays after electrophoresis on 1.2% agarose gel in a Tris-acetate EDTA buffer stained with ethidium bromide for 45 min [8].

### 2.7. Nucleotide Sequencing, Alignment, and Phylogenetic Analysis

The VP1-amplified PCR bands (814 bp) were purified from the agarose gel using a gel purification kit (Qiagen, MD, USA) and sequenced in an ABI3730xl Genetic Analyzer (Applied Biosystems, Foster City, CA, USA) using an ABI PRISM Big Dye Terminator v 3.1 Ready Reaction Cycle Sequencing Kit (Thermo Fisher, Franklin, MA, USA). The same forward and reverse specific primers for the previous PCR were used. The obtained sequences in both directions (forward and reverse) were checked together, and multiple nucleotides and deduced amino acid sequences of the isolates were aligned together using the MEGA11 software [31]. A maximum likelihood (ML) phylogenetic tree was constructed using the Tamura 3-parameter model by MEGA11 software [31,32]. This tree was based on the 600 bp sequence of VP1, corresponding to the nucleotide positions 2364–2964 in the whole genome sequence of the KP940474/A/Egypt/2014 reference strain. It included two isolates out of the obtained identical FMDV sequences, together with the 30 sequences from the NCBI nucleotide blast (https://blast.ncbi.nlm.nih.gov/Blast.cgi (accessed on 1 July 2022)) that was reported previously in Egypt and worldwide.

### 2.8. Statistical Analysis

The morbidity and mortality rates were calculated via counting. An online statistics software http://vassarstats.net/ (accessed on 10 October 2022) was used to estimate the 95% confidence interval (CI) and Fisher Exact Probability Test’s (two-tailed) significance on the differences between the tested buffalo and cattle calves. A *p* value of <0.05 was considered statistically significant.

## 3. Results

### 3.1. Propagation of FMDV and Characterization of the CPE on BHK-21 Cells

FMDV propagation was demonstrated daily for the CPE on BHK-21 cells for three days and for three successive passages. BHK-21 cells in all inoculated flasks with the 20 supernatants from the infected calves were rounded in shape, sloughed, and died after the first day of inoculation in all passages, compared to spindle and attached confluent cells without remarkable changes in the negative control flask.

### 3.2. Molecular Detection and Serotyping of FMDV

FMDV was detected in all 20 RNA samples of inoculated third passages with tongue epithelium from the 20 infected calves. The positive results were indicated via the amplification of 328 bp from the 3D segment of the viral genome. For FMDV serotyping, the 20 positive samples were tested using serotype-specific primer sets which amplified 814, 1124, and 666 bp of the 1D variable gene of serotypes A, O, and SAT2, respectively. All 20 samples were positive for serotype A and negative for serotypes O and SAT2.

### 3.3. Nucleotide Sequences of FMDV, Serotype A

The serotype A amplicons were purified and sequenced on both sides (forward and reverse) using the same primers as the serotyping RT-PCR. The obtained sequences were edited and 600 nucleotides of the VP1 region were aligned together. The result revealed that the nucleotides and amino acid sequences of the 20 obtained isolates were identical for the VP1 region. Therefore, out of them, four identical isolates were deposited in the GenBank (https://www.ncbi.nlm.nih.gov/ (accessed on 10 November 2022)), with the accession numbers OP823161 (https://www.ncbi.nlm.nih.gov/nuccore/OP823161 (accessed on 27 November 2022)) and OP823162 for buffalo calves’ isolates, and OP823163 and OP823164 for cattle calves’ isolates.

### 3.4. Phylogenetic Analysis

**Figure 2:**
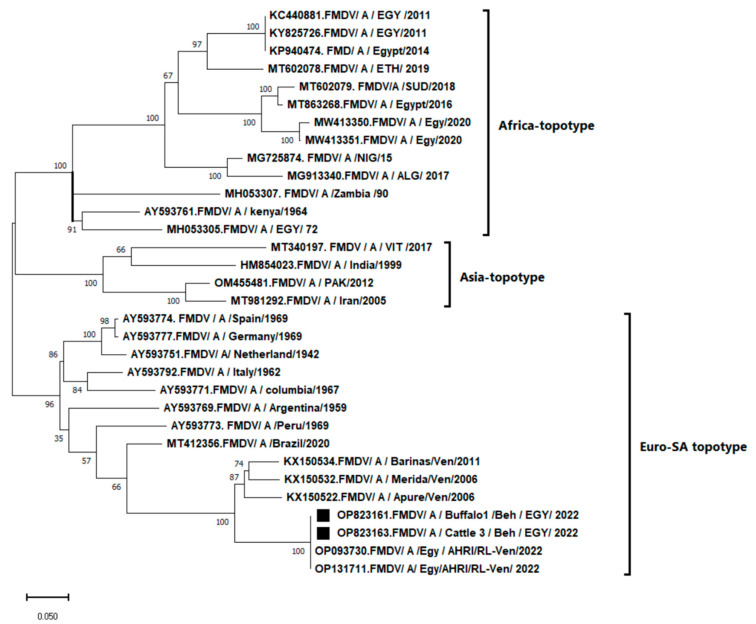
Maximum likelihood (ML) phylogenetic tree based on the 600 base pairs nucleotide of the VP1 region of foot-and-mouth disease virus genome corresponding to nucleotide positions 2364-2964 in the whole genome sequence of KP940474/A/Egypt/2014 reference strain. It included two isolates that were marked with black squares (accession No. OP823161 and OP823163) out of the 20 identical sequences in the current study, together with 30 isolates from GenBank (NCBI). These 30 isolates represented the different serotype A topotypes (Europe–South America, Asia, and Africa) that occurred worldwide, and each isolate was labeled with its accession number and the country of origin. Evolutionary analysis was conducted using MEGA11 software. One thousand replications were performed to calculate the bootstrap values indicated on the tree. The bar in the lower-left corner of the figure denotes distance.

A phylogenetic tree was constructed and included two of the obtained sequences and 30 sequences of serotype A that resulted from the nucleotide blast of NCBI. The constructed ML phylogenetic tree placed the virus within the Euro–SA topotype (Figure 2). The obtained isolates (accession No. OP823161-OP823164) were identical and shared 100% identity to the Euro–SA lineage (accession No. OP093730 and OP131711) that were reported recently by the Animal Health Research Institute, Egypt in 2022. The obtained isolates were closely related to the isolates from Venezuela (accession No. KX150534, KX150532, and KX150522) and shared 87.5%, 86.9, and 86.4% identity, respectively (Table 2). The obtained isolates shared the lowest nucleotide similarities with the previously reported serotype A, African topotype isolates from Egypt such as MH053305, MT86368, MW413351, MW413350, KP940474, KY825726, and KC440881 (Table 2). They also shared 66.8%, 66%, 65.3%, and 64.6% similarity with serotype A, Asian topotype isolates from Pakistan, Iran, India, and Vietnam, respectively (Table 2).

### 3.5. Amino Acid Sequences of the Immunogenic (G-H) Loop of the VP1 Region in the Obtained Strain Compared to FMD Vaccinal Strains Used in Egypt

**Figure 3:**
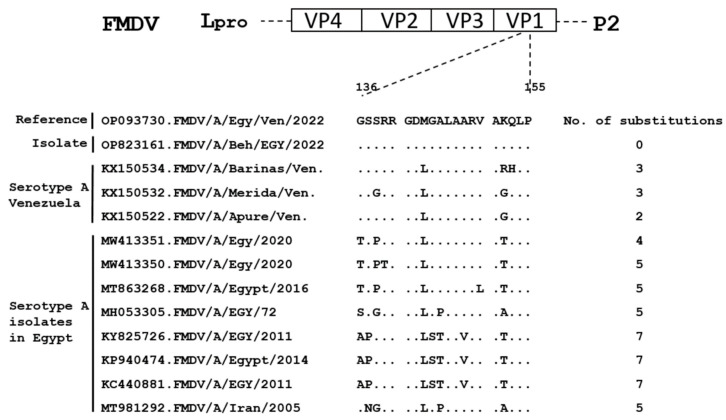
Comparison of amino acid sequences of the immunogenic (G-H loop) region of VP1 in the obtained isolate (accession No. OP823161) with the strain recently identified in Egypt (Reference), and isolates from Egypt, Venezuela, and others included in the Egyptian FMD vaccines. Each isolate was labeled with its accession number and the country of origin (Left panel). Alignment of amino acid sequences, located at 136–155 positions of G-H loop corresponding to amino acids positions 141–160 of whole VP1 sequence; the dots indicate the identity of the amino acids (Middle panel). The number of amino acid substitutions was counted and written in front of each isolate (Right panel).

The amino acid substitutions in the immunogenic (G-H) loop of the VP1 region in the obtained isolate (accession No. OP823161) were compared to the Euro–SA isolates from Egypt (accession No. OP093730) and Venezuela (accession No. KX150534, KX150532, and KX150522), whereas 0, 3, 3, and 2 amino acid substitutions were shown, respectively (Figure 3). More substitutions have been detected in Egyptian isolates (accession No. MW413351, MW413350, MT863268, MH053305, KY825726, KP940474, and KC440881) and revealed 4, 5, 5, 5, 7, 7, and 7 amino acid substitutions, respectively (Figure 3). The Asian, Iran05 isolate (accession No. MT981292) showed 5 amino acid substitutions (Figure 3).

### 3.6. The Epidemiological Patterns and Clinical Findings Related to the FMD Outbreak in One Infected Farm

Epidemiologically, all 70 calves (35 buffalo and 35 cattle) in the infected farm showed clinical signs of the disease with a morbidity rate of 100%. In total, 12 (17.1%) calves died. However, the mortality rates were 25.7% (9/35; 95% CI: 13–43) and 8.6% (3/35; 95% CI: 2–24; *p* = 0.11) in buffalo and cattle calves, respectively (Table 3). As reported by the veterinarians, death has been reported in a peracute form in buffalo calves (within 2–3 days), meanwhile, it took a longer time in cattle calves (6–8 days); whereas the course of the disease in recovered cases was 2–3 weeks in buffalo calves and 2–4 weeks in cattle calves (Table 3).

Clinically, the infected calves showed a wide range of signs. A full 100% of buffalo calves and over 77.1% (*p* = 0.004) of cattle calves experienced loss of appetite or anorexia. Buffalo calves more frequently exhibited dullness (85.7; 95% CI: 69–95) than cattle calves (62.9%; 95% CI: 45–78; *p* = 0.054), while the greatest difference was in observed recumbence or preferred recumbence which affected 71.4% (95% CI: 53–85) of buffalo calves but only 25.7% (95% CI: 13–43; *p* = 0.0002) of cattle calves (Table 3). The prominent FMD signs, such as vesicles on the mouth and foot, salivation, and lameness, have been reported in 65.7% (95% CI: 51–84), 40% (95% CI: 24–58), and 25.7% (95% CI: 13–43) of buffalo, compared to 88.6% (95% CI: 72–96; *p* = 0.04), 71.4% (95% CI: 53–85; *p* = 0.01), and 34.3% (95% CI: 20–52; *p* = 0.6) of cattle calves, respectively (Table 3). In total, 70 (100%) calves suffered from abnormal rectal body temperatures of 36.2–41.5 °C and 36.5–41.7 °C for buffalo and cattle calves, respectively. Hyperthermia was recorded in 65.7% (95% CI: 51–84) and 88.6% (95% CI: 72–96; *p* = 0.04), whereas hypothermia was recorded in 34.3% (95% CI: 20–52) and 11.4% (95% CI: 4–28; *p* = 0.04) of infected buffalo and cattle calves, respectively (Table 3).

Heart rates were counted via stethoscope in diseased calves (38–160 and 39–155 beats per min) of buffalo and cattle calves, respectively. Tachycardia, bradycardia, and cardiac arrhythmia were reported in 65.7% (95% CI: 51–84), 34.3% (95% CI: 20–52), and 40% (95% CI: 24–58) of buffalo, compared to 88.6% (95% CI: 72–96; *p* = 0.04), 11.4% (95% CI: 4–28; *p* = 0.04), and 17.1% (95% CI: 7–34; *p* = 0.06) of cattle calves, respectively (Table 3). Respiratory cycles per minute were counted for each calf (11–62 and 12–66) of buffalo and cattle, respectively. Abnormal lung sounds and nasal discharges were found in 45.7% (95% CI: 29–63) and 22.9% (95% CI: 11–41) of buffalo calves, respectively, compared to 80% (95% CI: 63–91; *p* = 0.005) and 57.1% (95% CI: 40–73; *p* = 0.006) of cattle calves, respectively (Table 3). Some chronic clinical signs that were not reported prior to the FMD outbreak have occurred. For example, out of the 58 recovered calves, 2 (7.7%; 95% CI: 1–26) buffalo calves compared to 4 (12.5%; 95% CI: 4–30; *p* = 0.6) cattle calves showed chronic respiratory signs, and only 2 (7.7%; 95% CI: 1–26; *p* = 0.19) buffalo calves suffered from chronic diarrhea (Table 3).

## 4. Discussion

Egypt has a bovine population of about 5.1 million heads [33], with the highest density located in the Beheira province [34]. This study proved the existence of the recently identified [14] and newly emerging FMDV, serotype A, Euro–SA in the calves of Beheira. Furthermore, it was the first to investigate the effects of this strain on the Egyptian buffalo and cattle calves, as summarized in Table 4.

In the last few years, several studies have investigated the circulating FMDV serotypes in different localities of Egypt [8,9,13,14,20,21,35,36,37,38,39]. However, since the detection of the SAT2 serotype in 2018 [21], the circulating serotypes in Beheira have never been investigated. This study indicated the circulation of the newly emerging serotype A, Euro–SA lineage in all infected farms under investigation. This strain had been first identified in Egypt in 2022 [14]. The evolution analysis of the obtained isolates revealed a close genetic relatedness to the Venezuelan isolates. There is a long history of using virus genetic data to study the international spread of FMDV [40]. In accordance with our study, the majority of studies regarding FMDV genomics are limited to VP1, as it contains serotype-specific amino acid sequences differentiating various serotypes, topotypes, and lineages [3,41,42,43,44]. In contrast, other researchers were unable to use this method to differentiate between the subtypes as there were no substitutions in VP1 sequences at the time [8].

Globalization of the FMDV spread between countries is associated with the movements of people, goods, animals, and their products. Unrestricted and illegal movements of animals are especially risky [45]. As Egypt imports live animals from several countries [34], Egypt act as a bridge for FMDV transmission between countries [12].

FMDV in South America circulates serotype A, topotype Euro–SA and serotype O, topotype Euro–SA as sporadic outbreaks in Venezuela, Colombia, and Ecuador [12,46,47]. Although the South American nations established a plan (2011–2020) for the eradication of FMD through mass vaccination of animals via 700 million doses administered annually [48], Colombia had reported the occurrence of FMD outbreaks near the borders of Venezuela in 2017 and 2018 [49]. Then, Colombia successfully eradicated such infections and regained its freedom status [14]. Surprisingly, Egypt had reported two new emerging outbreaks in 2022 related to the South American isolates [13,14]. This endorses that animal movement across international borders predisposes Egypt to newly emerging FMDV serotypes [50]. Furthermore, the genetic and antigenic relatedness of commercially used inactivated FMDV vaccinal strains and the Egyptian-circulating viruses are unknown. Therefore, huge economic losses are associated with newly emerging FMDV strains [20,26].

In this context, we compared the amino acids sequences of the immunogenic site G-H loop (residues 141–160) of VP1 [51,52] in the obtained serotype A isolates from Egypt, Venezuela, and Asia. Some of these isolates were included in the vaccines produced by the Veterinary Serum and Vaccine Research Institute in Egypt. The results suggested that routine identification of the circulating viruses in order to control the disease via vaccination is necessary [53]. Up to 2021, Egypt was using an inactivated local trivalent vaccine (containing O Pan-Asia 2, A Iran/05, and SAT2 Ghb-12 lineage seeds) in combination with a monovalent vaccine containing SAT2 Lib-12 lineage seeds for the mass vaccination of susceptible animals. Alternatively, some farmers were using an imported hexavalent vaccine (containing O Manisa, O-3039, A Iran 05, A Saudi 95, SAT2 Eritrea, and Asia1 Shamir seeds) [20]. Since July 2022, a new polyvalent FMD inactivated vaccine has been produced by VSVRI and contains O, A Iran05, A Africa 2020, SAT2-2012, A Africa G IV Egypt 2022, and A-Venezuela (http://vsvri.com/Products.html (accessed on 20 December 2022)).

In the current study, we compared the epidemiological features and clinical parameters related to such FMDV strains in buffalo and cattle calves (4–6 months old). On the semi-intensive farm level, all buffalo and cattle calves were infected. However, the mortality rate was significantly higher (25.7%) and in peracute form (within 2–3 days) in buffaloes compared to cattle calves (8.6% died within 6–8 days). These findings were in accordance with the theory that the FMDV spread in a susceptible population approaches 100% with high mortalities in young animals [4,7,8]. For example, Mahmoud and Neamat-allah [54] reported 100% mortality among infected suckling buffalo calves from Sharquia with a tiger heart appearance caused by serotype SAT2. On the other side, Hefnawy and colleagues reported a lower virulence in vaccinated buffalo calves in the Menofia province associated with serotype O infection [9]. Regarding cattle calves, the infected cattle calves in the current study showed a lower mortality rate than that caused by the previous FMDV serotypes of A and O [8]. However, Abd El Moneim and colleagues reported 93.7% of mortalities in suckling cattle calves in Dakahlia were caused by serotype A [55]. The previous studies in Egypt linked the mortalities among buffalo and cattle calves to myocarditis [8,9,38,54].

In young calves, myocarditis is the main cause of death during FMD outbreaks that may occur without the appearance of the characteristic vesicular lesions [1,9]. Our results revealed that the high and rapid fatal cases in buffalo calves were associated with hypothermia, bradycardia, and cardiac arrhythmia. However, a higher number of recovered cases in cattle calves was associated with tachycardia and rhythmic heartbeats. Furthermore, abnormal lung sounds, nasal discharges, and respiratory signs have been reported in cattle calves at a higher rate than buffalo calves, even in the chronic cases that recovered from FMD symptoms. Theoretically, FMD signs can appear within 2 to 3 days after exposure and can last for 7 to 10 days. A number of studies have suggested that the lung or pharyngeal areas are the sites of initial virus replication in cattle, with the rapid dissemination of the virus to oral and pedal epithelial areas [15]. Moreover, pneumonia and pulmonary lesions usually follow systemic infection and thrombosis of the lung after FMD infection [55]. Additionally, cattle can develop a rapid and vigorous genuine local immune response throughout the respiratory tract after/with FMD infection [56]. Notably, more than 50% of cattle that recover from FMD can become carriers with low levels of infectious virus in their pharyngeal region for a long period of time. In this case, the possibility of transmission of the disease from carrier cattle to naïve animals under controlled conditions is limited [57].

This study had two limitations. Firstly, it was carried out with a small number of samples from only five infected farms. Secondly, epidemiology and clinical findings related to such a newly emerging variant were carried out on a single infected farm. Consequently, this might broaden the CI range in mortality measurements that are used to compare cattle and buffaloes. However, as all animals used were kept on the same farm and were subjected to the same breeding, nutritional, and management conditions, the confounding effects of these factors on the disease outcome are highly reduced; therefore, we believe that the observed differences between the cattle and buffaloes are the direct result of FMDV infection. We believe that a comprehensive study including a higher number of farms could shed more light on the disease’s epidemiology and clinical picture in calves.

## 5. Conclusions

This study molecularly detected FMDV, serotype A, Euro-SA lineage in outbreaks in different localities of the Beheira province, where it has never been previously reported. This strain was newly emerging in Egypt, as published by the Animal Health Research Institute in July 2022. We showed a high polymorphism in the amino acid sequences of the immunogenic region (G-H loop) compared to the vaccinal strains used in Egypt. High morbidity was reported among young calves with higher peracute mortalities and more severe clinical findings in buffalo than in cattle calves. Further study is in progress to investigate the causes of higher mortality rates in buffalo than in cattle calves observed with this newly emerging FMDV variant. Additionally, a large-scale and continuous detection of FMDV variants circulating in the Beheira province is needed and the use of regularly updated vaccines is recommended.

## Figures and Tables

**Table 1:** Number and distribution of samples from cattle and buffalo calves in Beheira province.

	Location	Damanhour	Abu Hommus	Abu Almatamer	Kafr El-Dawar	Total
Population						
No. of tested farms		1	2	1 *	1	5
No. of samples per farm		4	8	8	4	20
No. of cattle/buffalo samples		2:2	4:4	2:2	2:2	10:10
History of FMD vaccination		No	No	No	Yes	1
Study of clinical and epidemiological parameters in farms		No	No	Yes	No	2

* Farm was comprising 70 calves that were investigated for epidemiological and clinical parameters.

**Table 2:** Nucleotides and deduced amino acids’ identity of the obtained isolates (OP823161–OP823164) compared to worldwide sequences of serotype A lineage.

Topotype	Accession No.	Country	Nucleotides Identity (%) *	Amino Acids Identity (%) **	Origin	Isolation Year
Euro-SA						
	OP823161	Egypt	Study isolate	-	Buffalo	2022
	OP823164	Egypt	Study isolate	-	Cattle	2022
	OP093730	Egypt	100	100	Cattle	2022
	OP131711	Egypt	100	100	Cattle	2022
	KX150534	Venezuela	87.5	87.6	Bovine	2011
	KX150532	Venezuela	86.9	86.9	Bovine	2006
	KX150522	Venezuela	86.4	89.5	Bovine	2006
	MT412356	Brazil	78.9	82.4	Bovine	2020
	AY593773	Peru	73.2	85.7	N. host ***	1969
	AY593774	Spain	72.7	80.1	N. host ***	1969
	AY593769	Argentina	72.2	79.5	N. host ***	1959
	AY593777	Germany	72.1	80.1	N. host ***	1969
	AY593751	Netherland	70.7	80.1	N. host ***	1942
	AY593771	Colombia	70.2	82.9	N. host ***	1967
	AY593792	Italy	70	80.1	N. host ***	1962
Asia						
	OM455481	Pakistan	66.8	77.3	Buffalo	2012
	MT981292	Iran	66	78.8	Cattle	2005
	HM854023	India	65.3	78.8	Cattle	1999
	MT340197	Vietnam	64.6	78.5	Cattle	2017
Africa						
	AY593761	Kenya	70.3	79.5	N. host ***	1964
	MH053305	Egypt	67.5	80.2	Cattle	1972
	MG725874	Nigeria	65	75	Cattle	2015
	MH053307	Zambia	64.6	77.3	Cattle	1990
	MT863268	Egypt	63.2	74.2	Buffalo	2016
	MT602078	Ethiopia	62.3	76.5	Cattle	2019
	MW413351	Egypt	62.2	73.4	Cattle	2020
	MT602079	Sudan	61.4	73.4	Cattle	2018
	MW413350	Egypt	59.8	70.1	Cattle	2020
	MG913340	Algeria	59.7	72.6	Cattle	2017
	KP940474	Egypt	58.6	72.6	Cattle	2014
	KY825726	Egypt	58.6	72.6	N. host ***	2011
	KC440881	Egypt	58.6	72.6	Bovine	2011

* Partial nucleotide sequence of 600 bp of 1D gene. ** A number of 200 deduced amino acids. *** Isolates obtained from their natural hosts and adapted to guinea pigs or cell lines.

**Table 3:** Differences in epidemiological parameters and clinical findings of FMD-infected buffalo and cattle calves in a mixed-rearing Egyptian farm.

Epidemiological & Clinical Findings	Infected Buffalo Calves (%; 95% CI)	Infected Cattle Calves (%; 95% CI)	*p*-Value *	Total (%)
**No. of tested calves**	35	35		70
**Epidemiological parameters**				
Morbidity rate	35/35 (100.0)	35/35 (100.0)		70/70 (100.0)
Mortality rate	9/35 (25.7; 13–43)	3/35 (8.6; 2–24)	0.11	12/70 (17.1)
Case-fatality rate	9/35 (25.7; 13–43)	3/35 (8.6; 2–24)	0.11	12/70 (17.1)
Course of disease in fatal cases	2–3 days	6–8 days		2–8 days
Course of disease in recovered cases	2–3 weeks	2–4 weeks		2–4 weeks
**Clinical findings**				
Inappetence to anorexia	35/35 (100.0)	27/35 (77.1)	0.004	62/70 (88.6)
Dull and depressed	30/35 (85.7; 69–95)	22/35 (62.9; 45–78)	0.054	52/70 (74.3)
Recumbent or prefer recumbence	25/35 (71.4; 53–85)	9/35 (25.7; 13–43)	0.0002	34/70 (48.6)
Vesicles on mouth and foot	23/35 (65.7; 51–84)	31/35 (88.6; 72–96)	0.04	54/70 (77.1)
Salivation	14/35 (40.0; 24–58)	25/35 (71.4; 53–85)	0.01	39/70 (55.7)
Lameness	9/35 (25.7; 13–43)	12/35 (34.3; 20–52)	0.6	21/70 (30.0)
**Clinical parameters**				
Body temperature				
Rectal temperature range (°C) ^b^	36.2–41.5 ^a^	36.5–41.7 ^a^		-
Abnormal temperature	35/35 (100.0)	35/35 (100.0)		70/70 (100.0)
Hyperthermia	23/35 (65.7; 51–84)	31/35 (88.6; 72–96)	0.04	54/70 (77.1)
Hypothermia	12/35 (34.3; 20–52)	4/35 (11.4; 4–28)	0.04	16/70 (22.9)
**Heart auscultation**				
Heart rate range (Beat/minute) ^c^	38–160 ^a^	39–155 ^a^		-
Tachycardia	23/35 (65.7; 51–84)	31/35 (88.6; 72–96)	0.04	54/70 (77.1)
Bradycardia	12/35 (34.3; 20–52)	4/35 (11.4; 4–28)	0.04	16/70 (22.9)
Cardiac arrhythmia	14/35 (40.0; 24–58)	6/35 (17.1; 7–34)	0.06	20/70 (28.6)
**Respiratory parameters**				
Respiratory rate (Resp. cycles/minute) ^d^	11–62 ^a^	12–66 ^a^		-
Abnormal lung sound	16/35 (45.7; 29–63)	28/35 (80.0; 63–91)	0.005	44/70 (62.9)
Nasal discharge	8/35 (22.9; 11–41)	20/35 (57.1; 40–73)	0.006	28/70 (40.0)
**Chronic signs in recovered calves**				
Respiratory signs	2/26 (7.7; 1–26)	4/32 (12.5; 4–30)	0.6	6/70 (8.6)
Diarrhea	2/26 (7.7, 1–26)	0	0.19	2/70 (2.9)

^a^ Rectal temperature, heart rate, and respiratory rate ranges of all 35 diseased calves. ^b^ Normal temperature of buffalo calves is 38–38.8 °C and cattle calves is 37.9–38.7 °C. ^c^ Normal heart rate of buffalo calves is 90–110 and cattle calves is 95–112 beats/min. ^d^ Normal respiratory rate of buffalo calves is 20–25 and cattle calves is 19–28 beats/min. * *p*-value of <0.05 was considered statistically significant.

**Table 4:** Summary of the effect of the identified FMD, serotype A, Euro–SA on buffalo and cattle calves during an outbreak in the Beheira province.

	Animal	Buffalo Calves	Cattle Calves
Item			
Causative agent		FMD, Serotype A, Euro-SA	FMD, Serotype A, Euro-SA
Cytopathic effect		Observed on 1st day	Observed on 1st day
VP1 gene similarity		Identical to OP093730	Identical to OP093730
Morbidity		High	High
Acute clinical signs		More severe	Severe
Chronic signs		Yes	No
Fatality		Rapid and high	Slow and less

## Data Availability

The nucleotide sequence data used in this study are available on GenBank, National Library of Medicine, (NCBI) under accession numbers OP823161, OP823162, OP823163, and OP823164.
